# Prevalence and Risk Factors for Malnutrition in Patients With Parkinson's Disease

**DOI:** 10.3389/fneur.2020.533731

**Published:** 2020-12-10

**Authors:** Tianting Yang, Zhen Zhan, Liang Zhang, Jun Zhu, Yi Liu, Lili Zhang, Jianchao Ge, Ying Zhao, Li Zhang, Jingde Dong

**Affiliations:** Nanjing Brain Hospital Affiliated to Nanjing Medical University, Nanjing, China

**Keywords:** Parkinson's disease, malnutrition, MNA, disease severity, dyskinesia

## Abstract

**Objectives:** This study aimed to investigate the relationship between nutritional status and Parkinson's disease (PD) features.

**Methods:** The cohort was composed of 556 Parkinson's patients who were admitted to the hospital. Patients were categorized as normal nutrition or at risk of malnutrition/already malnourished. Questionnaires, physical examinations, and biochemical tests were conducted. The relationship between nutrition status and PD was analyzed using *t*-tests, χ^2^-tests, and logistic regression models.

**Results:** The prevalence of malnutrition [defined as a Mini Nutritional Assessment (MNA) score <17] was 39.2%, and 30.3% of patients were at risk of malnutrition (17 ≤ MNA score ≤ 23.5). There was no difference in gender and age between the different nutrition groups (*P* < 0.05). Patients at risk of malnutrition and those who were malnourished had a longer course of disease, more severe motor symptoms, a higher stage of PD according to the Hoehn and Yahr (H-Y) classification, a lower body mass index (BMI) index, a lower cognitive score, higher levels of depression and anxiety, and more serious non-motor symptoms (*P* < 0.05) than patients with normal nutrition. There were differences in adenosine deaminase, albumin, phosphorus, chlorine, total protein, and uric acid between the two groups (*P* < 0.05). High Unified PD Rating Scale (UPDRS-III) scores, high H-Y stages, and dyskinesia were risk factors for malnutrition in PD patients, while high levels of total protein, uric acid, and chlorine were protective factors that led to good nutrition (*P* < 0.05).

**Conclusions:** Our results showed that dyskinesia, disease severity, total protein levels, uric acid levels, and chlorine levels were associated with nutritional status among Chinese PD patients. The findings of this study indicate the significance of the early detection and prevention of malnutrition to improve the quality of life of PD patients.

## Introduction

Parkinson's disease (PD) is a chronic degenerative neurodegenerative disease. The incidence of PD is ~2% among individuals over 65 years old in China, accounting for more than 40% of the total number of PD patients in the world. The prevalence increases annually. PD seriously endangers the physical and mental health of elderly people and causes huge economic and social burdens (1).

Some studies have shown that some lifestyle interventions could improve the quality of life of PD patients, such as behavior therapy, sleep improvement, mood improvement, and physical exercise (2–5). However, few studies have examined the effect of nutritional status on the quality of life of patients with PD (6–8). A study showed a negative association between the severity of motor impairment and total fat mass in patients with PD (7). Another study showed that malnutrition in PD patients can cause olfactory and gustatory dysfunction (9). Nutritional risk screening plays an increasingly important role in PD. Therefore, we evaluated the cross-sectional association between nutritional status and PD to better predict the prognosis and development of PD.

## Materials and Methods

### Population

A consecutive series of 556 hospitalized PD patients (324 men and 232 women) from the Brain Hospital Affiliated to Nanjing Medical University during the period January 2015–December 2019. These participants conformed to the diagnostic criteria of UK Brain Bank PD, and we excluded patients with other diseases, such as serious heart disease, liver disease, dementia, and kidney disease. Moreover, all participants with confirmed digestive-system diseases or other consumptive diseases, such as infections and tumors, were excluded from this nutritional assessment. This study was approved by the Ethics Committee of the Brain Hospital Affiliated to Nanjing Medical University. We obtained informed consent from the patients.

### Procedures

All participants were divided into three groups based on nutritional status. Questionnaires, physical examinations, and biochemical tests were conducted. The Mini Nutritional Assessment (MNA) is a short valid nutritional screen for elderly people that is recommended by the European Society for Clinical Nutrition and Metabolism (ESPEN) (10). The MNA was used in this study to categorize patients as normal nutritional status, risk of malnutrition, or already malnourished. The final score predicts malnutrition (MNA score 24–30, normal nutritional status; MNA score 17–23.5, at risk of malnutrition; and MNA score <17, already malnourished) (11). The Unified PD Rating Scale part III (UPDRS-III), Hoehn and Yahr (H-Y) stage, and PD Non-Motor Symptoms (PD-NMS) questionnaire were used to assess the motor symptoms, non-motor symptoms, and severity of PD. The Mini-Mental State Examination (MMSE) scale was used to evaluate the cognitive level of PD patients. The Hamilton Depression Rating Scale (HAMD) and Hamilton Anxiety Rating Scale (HAMA) were used to evaluate the depression and anxiety levels of PD patients, respectively. Patients were divided into tremor-dominant type and akinetic-rigid type PD subgroups using part III of the UPDRS (12). Participants were measured for height with a height meter and sitting height with a sitting height meter; they were required to take off shoes and caps before measurements, and the results were accurate to 0.1 cm. Additionally, body weight was measured with an electronic weighing machine; patients were required to wear only single clothes, and the results were accurate to 0.1 kg. The body mass index (BMI) was calculated by dividing body weight (kg) by the square of height (m^2^). Levodopa equivalent daily dose (LEDD) was calculated according to previously published recommendations (13). Participants were asked to fast, and then, at 6:00 a.m., a total of 5 ml of venous blood was collected for routine blood tests (automatic blood cell analyzer, Myry), biochemical tests, and blood lipid detection (biochemical analyzer, Olympus).

### Statistical Analysis

All statistical analyses were performed with SPSS software version 19.0 (SPSS Inc., Chicago, IL). Numerical data are presented as the mean ± SD. Differences between two groups were evaluated with Student's *t*-test for quantitative data. Differences between two groups were evaluated with the χ^2^-test for qualitative data. A multivariate analysis using a forward binary logistic regression model with nutrition as the dependent variable and the above significant disease characteristics [duration of the disease, UPDRS-III score, H-Y classification, dyskinesia, MMSE score, HAMA score, HAMD score, PD-NMS score, LEDD, albumin (ALB) level, total protein (TP) level, uric acid (UA) level, phosphorus (P) level, chlorine (CL) level, and adenosine deaminase (ADA) level] as independent covariables was used to explore the potential clinical factors that may be related to nutrition. As BMI and weight were closely associated with nutrition and the MNA assessment, they were not be considered in the logistic analysis. Differences were considered significant at *P* < 0.05.

## Results

### Nutritional Grading of PD Patients

A tabular description of the study population based upon the nutritional status of PD patients is presented in Table 1. Overall, 556 participants (324 men and 232 women, which accounted for 58.3 and 41.7%, respectively) aged 36–92 years from our hospital were included in this cross-sectional study. The duration of disease among these patients ranged from 0.3 to 30 years, with an average of 6.34 ± 4.80 years. The UPDRS-III scores of these patients ranged from 2 to 80, with an average of 25.02 ± 12.37. According to the MNA score, 171 patients had normal nutrition status, 167 patients were at risk of malnutrition and 218 patients were malnourished, which accounted for 30.8, 30.0, and 39.2% of the sample, respectively.

**Table 1:** Subject demographic and clinical features of PD patients.

	**Total (*N* = 556)**	**Range**
Sex (male %)	324 (58.3%)	–
Age, mean ± SD	68.37 ± 10.47	36–92
Duration of disease, years	6.34 ± 4.80	0.3–30
UPDRS-III, mean ± SD	25.02 ± 12.37	2–80
H-Y stage, mean ± SD	2.41 ± 0.73	1–5
Normal nutrition status	171 (30.8%)	–
At risk of malnutrition	167 (30.0%)	–
Malnourished	218 (39.2%)	–
Dyskinesia (%)	11.9%	–
Yes	66	–
No	490	–
PD type		–
Tremor-dominant	291	–
Akinetic-rigid	265	–
Body weight, kg, mean ± SD	59.72 ± 8.41	41–83
Body weight at disease onset, kg, mean ± SD	64.45 ± 7.45	45–91
BMI, mean ± SD	23.07 ± 3.33	17.3–30.1
MNA, mean ± SD	19.82 ± 2.18	5–30
MMSE, mean ± SD	27.61 ± 2.17	20–30
HAMA, mean ± SD	10.73 ± 7.57	0–36
HAMD, mean ± SD	12.85 ± 9.02	0–49
PD-NMS, mean ± SD	10.77 ± 5.07	0–14
LEDD, mg, mean ± SD	420.32 ± 231.52	0–850

*PD, Parkinson's disease; MNA, Mini Nutritional Assessment; UPDRS-III, Unified PD Rating Scale; H-Y, Hoehn and Yahr stage; NMS, Non-Motor Symptoms Questionnaire; MMSE, Mini-mental State Examination; HAMD, Hamilton Depression Rating Scale; HAMA, Hamilton Anxiety Rating Scale; LEDD, Levodopa equivalent daily dose*.

### Comparison of General Clinical Data of PD Patients Based on Nutritional Status

Table 2 presents the general clinical data of PD patients at risk of malnutrition, patients who were malnourished and patients with normal nutrition. We found that there was no difference in gender or age between the two groups (*P* > 0.05). However, patients at risk of malnutrition and those who were malnourished had a longer duration of disease, more severe motor symptoms, a higher stage of PD according to the H-Y classification, a lower BMI, a lower cognitive score, higher levels of depression and anxiety, and more severe NMS.

**Table 2:** The general clinical data of PD patients at risk of malnutrition, malnourished patients, and patients with normal nutrition.

	**At risk of malnutrition and malnourished (*N* = 385)**	**Normal nutrition (*N* = 171)**	***t***	P*
Sex (male %)	226 (58.7%)	98 (57.3%)	0.094	0.759^a^
Age, mean ± SD	68.61 ± 10.30	67.85 ± 10.85	0.781	0.435^b^
Duration of disease, years	7.38 ± 4.97	4.10 ± 3.48	8.804	**<0.001**^**b**^
UPDRS-III, mean ± SD	29.47 ± 11.34	15.01 ± 7.80	17.188	**<0.001**^**b**^
H-Y stage, mean ± SD	2.64 ± 0.73	1.88 ± 0.57	13.708	**<0.001**^**b**^
Dyskinesia (%)	16.9%	0.5%		
Yes	65	1	30.066	**<0.001**^**a**^
No	320	170		
PD type				
Tremor-dominant	208 (54.0%)	83 (48.5%)	1.430	0.121^a^
Akinetic-rigid	177 (46.0%)	88 (51.5%)		
Body weight, kg, mean ± SD	52.51 ± 10.23	63.43 ± 9.93	9.347	**<0.001**^**b**^
Body weight at disease onset, kg, mean ± SD	65.13 ± 9.17	63.96 ± 9.83	1.896	0.067^a^
BMI, mean ± SD	22.51 ± 3.26	24.31 ± 3.15	−6.137	**<0.001**^**b**^
MNA, mean ± SD	14.10 ± 2.14	26.71 ± 2.51	−45.286	**<0.001**^**b**^
MMSE, mean ± SD	27.37 ± 2.28	28.15 ± 1.78	−4.336	**<0.001**^**b**^
HAMA, mean ± SD	11.65 ± 7.89	8.64 ± 6.34	4.791	**<0.001**^**b**^
HAMD, mean ± SD	13.95 ± 9.37	10.40 ± 7.62	4.709	**<0.001**^**b**^
PD-NMS, mean ± SD	11.56 ± 5.03	9.01 ± 4.72	5.605	**<0.001**^**b**^
LEDD, mg, mean ± SD	438.23 ± 256.15	393.52 ± 243.80	2.749	**0.009**^**b**^

a *Chi-square test*.

b *T-test*.

* *Patients at risk of malnutrition and malnourished patients were compared with the normal nutrition group*.

*PD, Parkinson's disease; MNA, Mini Nutritional Assessment; UPDRS-III, Unified PD Rating Scale; H-Y, Hoehn and Yahr stage; NMS, Non-Motor Symptoms Questionnaire; MMSE, Mini-mental State Examination; HAMD, Hamilton Depression Rating Scale; HAMA, Hamilton Anxiety Rating Scale; LEDD, Levodopa equivalent daily dose*.

*Bold value means P < 0.05*.

### Comparison of Biochemical and Blood Indicators of PD Patients Based on Nutritional Status

We also presents the biochemical and blood indicators of PD patients at risk of malnutrition, malnourished patients, and patients with normal nutrition (Supplementary Table 1). The results showed that there were differences in ADA, ALB, P, CL, TP, and UA between the two groups (*P* < 0.05).

### Factors Independently Correlated With Malnutrition

**Figure 1:**
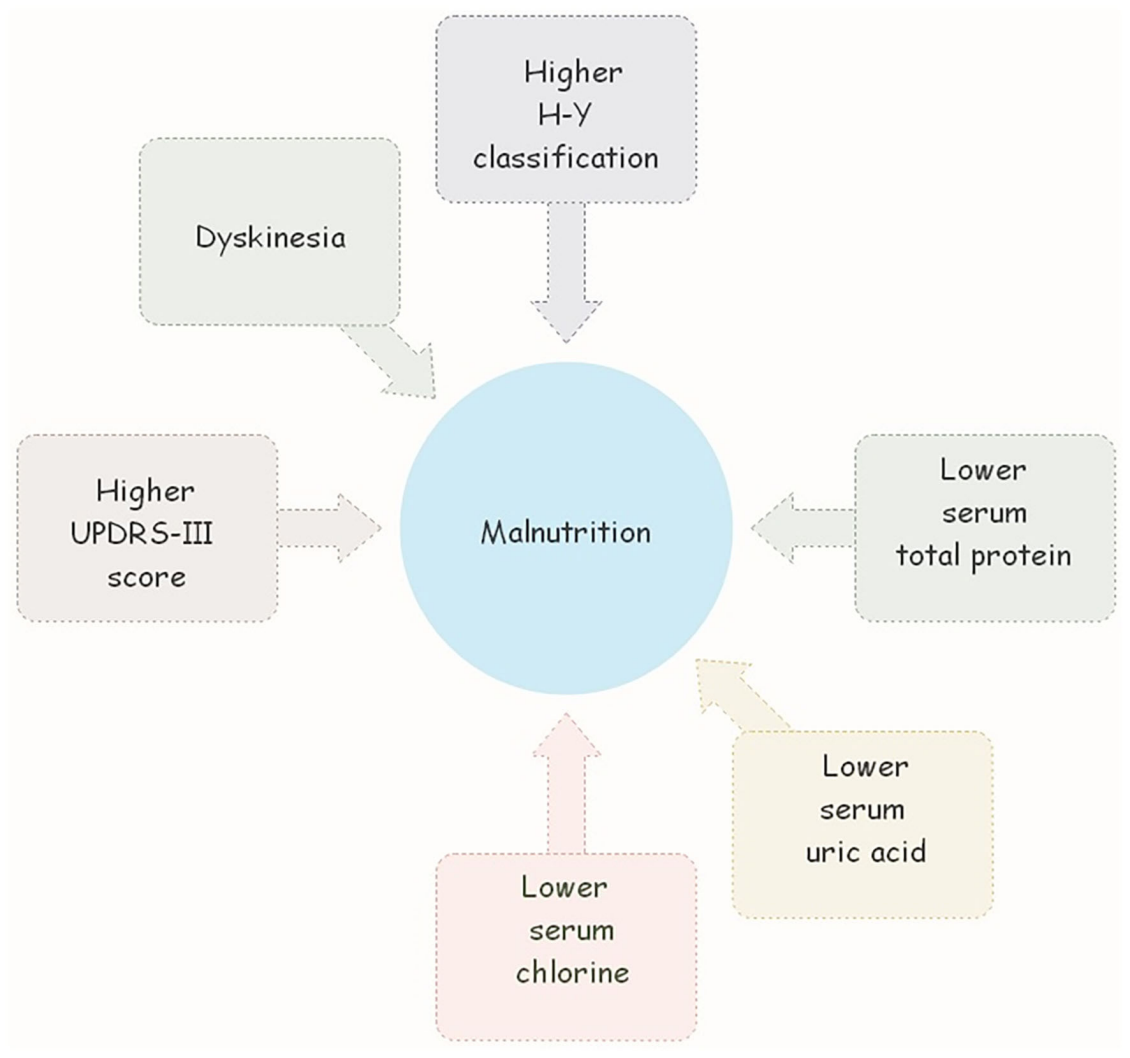
Higer UPDRS-III score, H-Y stage, dyskinesia, and the lower level of TP, UA, and CL were associated with nutritional status in PD.

Table 3 presents the multivariate analysis of associated factors for malnutrition in PD patients. The nutrition status was taken as the dependent variable (patients at risk of malnutrition and malnourished, patients with normal nutrition). The duration of the disease, UPDRS-III score, H-Y stage, dyskinesia, MMSE score, HAMA score, HAMD score, NMS score, LEDD, ALB level, P level, TP level, UA level, and ADA level were regarded as independent variables. Using the LR forward method, it was concluded that UPDRS-III score, H-Y stage, dyskinesia, and the level of TP, UA, and CL were associated with nutritional status in PD (Figure 1).

**Table 3:** Multivariate unconditional logistic regression analysis of risk factors for malnutrition.

	**OR**	**95% Cl**	***P*^**a**^**
UPDRS-III	1.136	1.008–1.443	0.029
H-Y stage	2.136	1.894–2.896	0.000
Dyskinesia	9.758	2.063–93.976	0.000
TP	0.958	0.914–0.996	0.006
UA	0.989	0.979–0.998	0.021
CL	0.876	0.748–0.913	0.002

*OR, odds ratio; CI, confidence interval; UPDRS-III, Unified PD Rating Scale; H-Y, Hoehn and Yahr stage; TP, total protein; UA, uric acid; CL, chlorine*.

a *The P-value is calculated from a forward binary logistic regression analysis*.

## Discussion

There are many methods for nutritional risk assessment. Previous studies have shown the risk of malnutrition by using the Short-Form Mini-Nutritional Assessment (MNA-SF), the Nutritional risk screening 2002 (NRS 2002) tool, and the Malnutrition Universal Screening Tool (MUST) (14). Cereda believed that the MNA nutrition screening scale was suitable for elderly patients in different institutions, such as hospitalization, outpatient clinics, and nursing homes. MNA could indicate nutritional risk before the laboratory indicators of malnutrition change and has a strong ability of diagnosis and prediction (15). Therefore, we used the MNA score as a nutritional risk assessment tool in this study. According to the data obtained from our study, 69.2% of our patients had an abnormal nutritional status. In our study, 167 (30.0%) patients were at risk of malnutrition, and 218 (39.2%) patients had malnutrition. These numbers were higher than those in many previous studies (16–18). The fact that the population assessed in our study generally consisted of low-income patients with lower education levels who resided in rural areas may explain the higher malnutrition rates. Due to their low income levels, nutritional intake mostly included carbohydrates.

Risk factors for poor nutrition in adults include older age, living alone, dementia, depression, gastrointestinal dysfunction (such as dysphagia, constipation, and slow gastric emptying), comorbidities and polypharmacy. In PD, these risk factors are more common than in age-matched controls. Factors that are specific to PD may include motor symptoms, NMS, older age at diagnosis, higher LEDD/body weight, depression, dementia, and hallucinations (19, 20). Limited research has been conducted to determine the predictors of malnutrition in Chinese PD patients.

In this study, we found that there were no differences in age and sex between patients at risk of malnutrition, malnourished, and normal nutrition. We also compared the general data, clinical characteristics, and biochemical indicators of the normal nutrition and malnutrition risk groups and found that the duration of disease, motor symptoms, stage of PD, BMI, cognitive scores, depression, and anxiety scores, NMS score, ADA level, ALB level, P level, Cl level, TP level, and UA level differed between the two groups. Then, to reduce bias caused by confounding factors, we used multivariate unconditional logistic regression analysis, which revealed that high UPDRS-III scores, a high H-Y stage, and dyskinesia were risk factors for malnutrition in PD patients, while high levels of TP, UA, and CL were protective factors that led to good nutrition.

Sheard et al. found that a greater UPDRS III score with a higher H&Y stage correlated with lower MNA results (20). We also found that malnourished patients had more pronounced motor symptoms and more severe disease stages. Decreased hand-mouth coordination and difficulties completing fine movements, such as that required for utensils, can be present. Motor symptoms such as bradykinesia, akinesia, rigidity, and tremor can impair functional ability and make it difficult to ambulate, shop, and feed independently.

In our patients with PD who had a poor nutritional status, higher levels of depression, and anxiety were observed. PD is often accompanied by anxiety and depression, which can influence the release of neurotransmitters and hormones in the body and cause intestinal dysfunction, constipation, and nutritional absorption disorders (21). The decrease in enthusiasm for daily activities in patients with PD due to retarded physical motions could also lead to reduced food intake. Reduced food intake, weight loss, and psychiatric disorders could lead to a vicious cycle.

We found that patients with dyskinesia were more likely to suffer from malnutrition, possibly because abnormal exercise leads to an increase in muscle nutrient consumption and eating difficulties. Energy expenditure may be increased by the presence of dyskinesia. Weight loss may result in an increased risk of developing dyskinesia that may, in turn, exacerbate weight loss and the risk of malnutrition.

Our results show that hyperuricemia is a sign of good nutrition, and the reasons may be as follows. Research shows that oxidative stress is a leading factor in the pathogenesis of idiopathic PD, and two intrinsic antioxidative molecules, i.e., bilirubin and UA, are known to protect dopaminergic neurons from oxidative stress in PD (22). Another study showed that the higher UA level group in female PD patients showed a smaller reduction in dopamine transporter uptake in the posterior putamen (23). However, it has been shown that hyperuricemia can also aggravate the development of PD (24). Therefore, how to balance the concentration of UA is a difficult problem at present.

CL is an important negatively charged ion (anion) used to maintain fluid balance. CL ions are the most abundant anions outside the cell. Together with sodium ions, CL ions account for ~80% of the total number of ions that maintain osmotic pressure. It can regulate the volume of extracellular fluid and maintain osmotic pressure. In addition, CL ions have the function of maintaining acid-base balance in body fluids, and excessive intake of CL ions can correct metabolic alkalosis caused by diseases or diuretics. Stomach acid promotes the absorption of vitamin B12 and iron, helps digest food, activates saliva amylase to breakdown starch, and inhibits the growth of microorganisms that enter the stomach with meals. In nerve cells, CL ions stabilize the membrane potential. The relationship between CL and nutrition is still unclear, especially in PD patients. Our research shows that serum CL is closely related to malnutrition. More research is needed to further clarify this correlation.

Adenosine, an important neuromodulator, is capable of attenuating oxidative stress, excitotoxicity, and neuroinflammation (25). On the other hand, adenosine is effective in promoting sleep, improving cognitive function and anti-depressive effects (26). These actions of adenosine have therapeutic implications for motor symptoms and NMS of PD. Several studies indicated that ADA inhibitors protected against neurodegeneration induced by the neurotoxin MPTP (27). In our study, there was a significant increase in ADA in patients at risk of malnutrition and malnourished patients. ADA was included in the logistic regression equation as an independent risk factor. However, ADA was not shown to be an independent predictor of malnutrition in PD patients. The relationship between ADA and nutrition is not fully understood. A study have analyzed if ADA could be considered a functional biochemical parameter in populations at nutritional risk. In this study, the serum ADA activity was analyzed in groups of individuals with altered nutritional status: young adult patients with Nervous Anorexia, overweight children and children suffering cystic fibrosis. The results showed ADA activity statistically significant increased in the in all groups, with respect to their healthy controls (28). The results suggested the importance of including the determination of serum ADA activity in the biochemical evaluation of the nutritional status. Further studies are needed to validate the relationship between ADA and nutrition in PD patients with longitudinal data.

There are many reasons for malnutrition. It was recently reported that the ketogenic diet is a low-carbohydrate and fat-rich diet. Its implementation has a fasting-like effect, which brings the body into a state of ketosis. Due to improper dietary control, PD patients have a ketogenic diet to reduce food intake and cause malnutrition (29). Therefore, we need to pay more attention to the dietary structure of PD patients. On the other hand, some studies have shown that vitamin D deficiency was significantly associated with the risk of PD and closely related to malnutrition in PD patients (30, 31). In future studies, we will pay more attention to the effects of vitamins and trace elements in PD patients to balance macro- and micronutrients. Additionally, owing to the different economic and cultural levels of different PD patients, some patients intake high levels of carbohydrates, which may lead to malnutrition. This phenomenon deserves our attention. We should provide good education and promote healthy nutrition among PD patients. Finally, most of the patients were enrolled from our hospital, so the sample may not be representative of the whole population. Additional general population studies are needed.

The strengths of this paper are the large sample and the evaluation of biochemical parameters. This study, however, has several limitations. It was a retrospective study, so it may be difficult to establish cause-effect associations. Longitudinal studies are needed in the future study. We have explained the selections of variables for the modes above. In the univariate analysis we found that patients with malnutrition showed had higher depression and anxiety scores, lower MMSE score, and a greater non-motor symptoms burden. However, neither of these non-motor symptoms emerged as an independent factor predictive of malnutrition in our study. Possible mechanisms underlying this comorbidity are difficult to interpret. It was a retrospective study, so it may be difficult to establish cause-effect associations. We are conducting a 5-year prospective, multicenter study on the nutritional status of Parkinson's patients. More advanced methods for testing the importance of these factors will be used in the future study.

## Conclusions

In summary, PD is a neurodegenerative disease, and malnutrition is its main concomitant symptom in the later stage. High UPDRS-III scores, high H-Y stages, dyskinesia, high levels of TP, UA, and CL were associated with nutritional status among Chinese PD patients. These findings provide evidence that nutritional status assessments should be standard during the treatment of PD patients.

## Data Availability Statement

The datasets generated for this study are available on request to the corresponding author.

## Ethics Statement

The studies involving human participants were reviewed and approved by Ethics Committee of Brain Hospital Affiliated to Nanjing Medical University. The patients/participants provided their written informed consent to participate in this study.

## Author Contributions

TY and JD participated in conception and design of the study, acquisition of raw data, analysis of data, drafting the article, and revising the manuscript. ZZ, LiaZ, JZ, and YL participated in conception and design of the study, collection of date, analysis of data, and revising the manuscript. LilZ, JG, YZ, and LiaZ participated in conception, design of the study, and revising the manuscript. All authors contributed to the article and approved the submitted version.

## Conflict of Interest

The authors declare that the research was conducted in the absence of any commercial or financial relationships that could be construed as a potential conflict of interest.
