# Supplementary material for: Prevalence and Risk Factors for Malnutrition in Patients With Parkinson's Disease
Source: Front Neurol. 2020 Dec 10;11:533731. doi: 10.3389/fneur.2020.533731 (PMC7758281; doi:10.3389/fneur.2020.533731)

Table S1. Comparison of hematological indicators in patients at the risk of malnutrition, malnourished and normal nutrition

|  | Total  （N=556） | At risk of malnutrition  and malnourished  （N=385） | Normal nutrition  （N=171） | t | P* ^a^ |
| --- | --- | --- | --- | --- | --- |
| HCT | 39.11±4.49 | 38.94±4.60 | 39.48±4.23 | -1.327 | 0.185 |
| HGB | 128.36±15.50 | 128.12±15.98 | 128.92±14.38 | -0.562 | 0.574 |
| MCH | 30.64±1.80 | 30.65±1.79 | 30.61±1.82 | 0.219 | 0.827 |
| MCV | 93.35±4.60 | 93.27±4.46 | 93.52±4.89 | -0.585 | 0.558 |
| MPV | 10.21±1.34 | 10.18±1.28 | 10.28±1.45 | -0.883 | 0.405 |
| PLT | 185.55±53.66 | 188.15±53.28 | 179.69±54.20 | 1.707 | 0.089 |
| RBC | 4.23±0.50 | 4.22±0.51 | 4.25±0.50 | -0.648 | 0.517 |
| WBC | 5.92±1.56 | 5.90±1.54 | 5.97±1.59 | -0.472 | 0.637 |
| ADA | 12.15±6.11 | 12.52±6.67 | 11.31±4.50 | 2.517 | **0.012** |
| ALB | 41.55±4.02 | 41.14±4.20 | 42.45±3.41 | -3.847 | **＜0.001** |
| GLO | 24.56±3.74 | 21.49±2.48 | 21.94±2.30 | -0.334 | 0.656 |
| ALT | 16.85±10.91 | 16.33±10.45 | 18.04±11.82 | -1.713 | 0.087 |
| AMY | 58.18±21.98 | 58.95±22.70 | 56.44±20.23 | 1.245 | 0.213 |
| Apo-A | 1.43±0.28 | 1.45±0.29 | 1.39±0.25 | 2.218 | 0.027 |
| Apo-B | 0.75±0.25 | 0.77±0.27 | 0.73±0.22 | 1.677 | 0.094 |
| Apo-E | 4.16±2.59 | 4.27±2.60 | 3.91±2.57 | 1.501 | 0.134 |
| AST | 24.26±13.06 | 24.54±13.07 | 23.63±13.06 | 0.762 | 0.447 |
| CHE | 289.59±74.27 | 286.64±74.57 | 296.23±71.04 | -1.405 | 0.160 |
| CHOL | 4.01±0.89 | 4.02±0.91 | 3.98±0.83 | 0.403 | 0.687 |
| CK | 82.63±35.90 | 82.50±35.75 | 82.94±36.34 | -0.135 | 0.893 |
| CL | 104.90±3.13 | 104.66±3.27 | 105.45±2.73 | -2.746 | **0.006** |
| CREA | 68.16±17.92 | 68.04±18.50 | 68.43±16.59 | -0.235 | 0.814 |
| DBIL | 4.72±2.08 | 4.77±2.16 | 4.61±1.89 | 0.819 | 0.413 |
| Fe | 14.99±6.34 | 14.79±6.20 | 15.45±6.63 | -1.128 | 0.260 |
| GLU | 5.18±1.08 | 5.17±1.12 | 5.20±0.98 | -0.327 | 0.743 |
| HDL | 1.27±0.30 | 1.27±0.32 | 1.25±0.25 | 0.813 | 0.417 |
| IBIL | 10.15±3.28 | 10.27±3.37 | 9.89±3.07 | 1.264 | 0.207 |
| K | 3.90±0.32 | 3.90±0.33 | 3.90±0.30 | -0.459 | 0.647 |
| LDH | 167.64±45.58 | 168.89±46.74 | 164.85±42.87 | 0.964 | 0.366 |
| LDL | 2.38±0.66 | 2.40±0.67 | 2.34±0.64 | 0.957 | 0.339 |
| Lpa | 130.45±105.53 | 129.49±108.77 | 132.62±98.12 | -0.322 | 0.747 |
| MG | 0.89±0.09 | 0.89±0.09 | 0.90±0.09 | -0.725 | 0.469 |
| NA | 140.23±2.89 | 140.08±2.89 | 140.57±2.88 | -1.861 | 0.063 |
| PA | 211.19±46.42 | 211.18±48.63 | 211.20±41.13 | -0.003 | 0.997 |
| P | 1.06±0.18 | 1.05±0.17 | 1.09±0.20 | -2.307 | **0.022** |
| RBP | 37.45±7.92 | 37.75±7.86 | 37.78±8.04 | 1.326 | 0.185 |
| TBA | 6.32±6.13 | 6.43±6.18 | 6.06±6.02 | 0.656 | 0.512 |
| TBIL | 14.90±5.09 | 15.07±5.22 | 14.52±4.75 | 1.168 | 0.243 |
| TG | 1.11±0.58 | 1.11±0.62 | 1.13±0.47 | -0.434 | 0.665 |
| TP | 62.30±6.01 | 61.70±6.31 | 63.65±5.00 | -3.909 | **＜0.001** |
| TRF | 2.08±0.30 | 2.09±0.30 | 2.07±0.30 | 0.441 | 0.659 |
| UA | 290.41±70.56 | 278.14±64.75 | 318. 04±75.33 | -39.890 | **＜0.001** |
| UREA | 5.50±1.79 | 5.56±1.94 | 5.36±1.40 | 1.370 | 0.171 |
| HCY | 18.85±11.69 | 19.48±10.18 | 18.16±10.96 | 0.914 | 0.426 |

a: T test

*：The risk of malnutrition or with malnutrition groups were compared with the normal nutrition

HCT: hematocrit, HGB: hemoglobin, MCH: mean corpuscular hemoglobin, MCV: mean corpusular volume, MPV: mean platelet volume, PLT: platelet count, RBC: red blood count, WBC: white blood cell count, ADA: adenosine deaminase, ALB: albumin, GLO：globulin, ALT: alamine aminotransferase, AMY: serum amylase, AST: aspartate transaminase, CHE: cholinesterase, CHOL: cholesterol, CK: creatine kinase, CL: chlorine, CREA: creatinine, DBIL: direct bilirubin, HDL: high density lipoprotein, IBIL: indirect bilirubin, MG: magnesium, LDL: low density lipoprotein, NA: sodium, P: phosphorus, RBP: retinol binding protein, TBA: total biliary acid, TBIL: total bilirubin, TG: triglyceride, TP: total protein, TRF: transferrin, UA: uric acid, HCY: homocysteinemia

Mini Nutrition Assessment


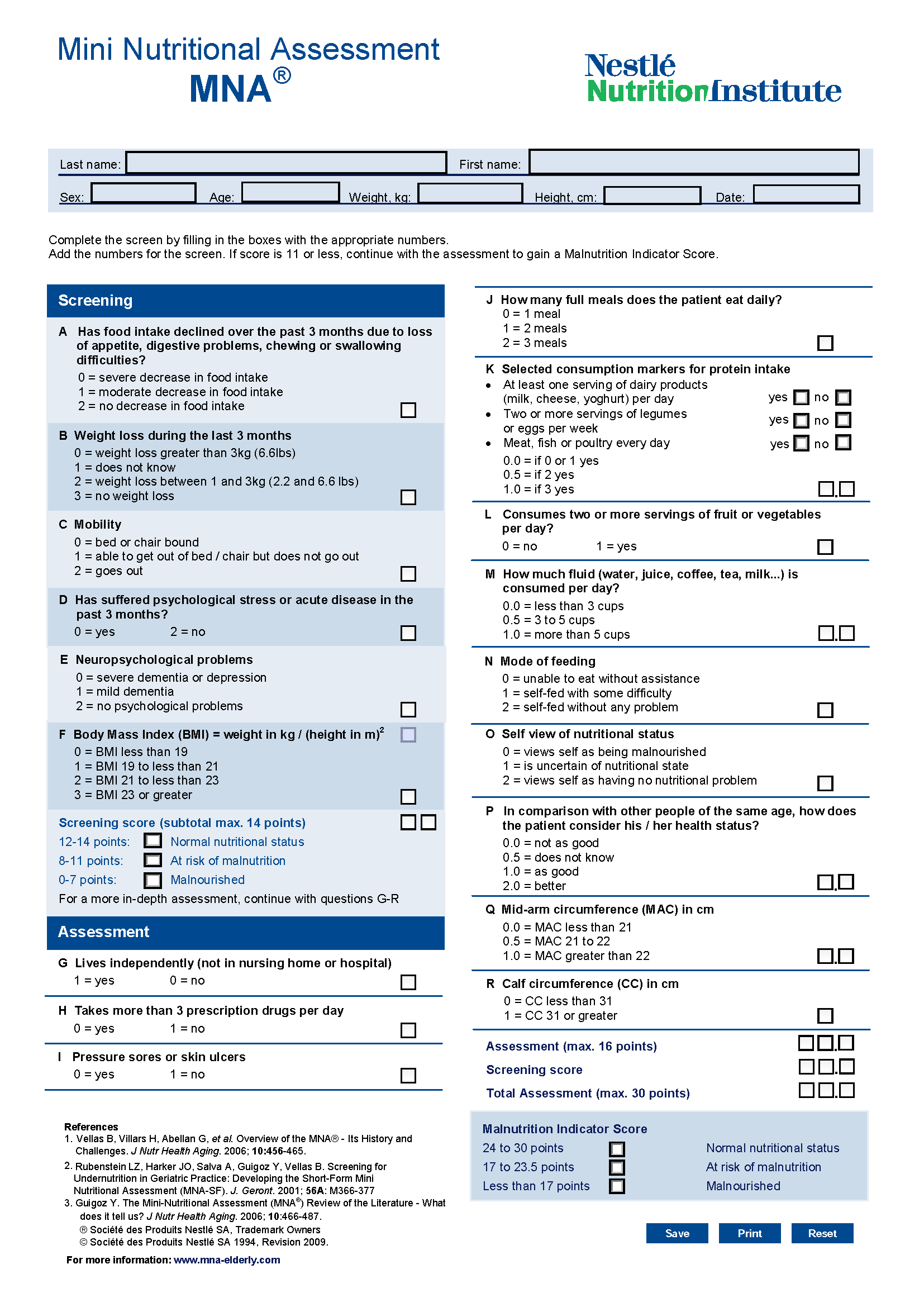

Supplement: Supplementary file 1 [file Table_1.doc]
